# Mesenchymal Stem Cell Therapy for Huntington Disease: A Meta-Analysis

**DOI:** 10.1155/2023/1109967

**Published:** 2023-04-30

**Authors:** Xue-Song Liang, Zheng-Wu Sun, Aline M. Thomas, Shen Li

**Affiliations:** ^1^Department of Neurology and Psychiatry, Beijing Shijitan Hospital, Capital Medical University, Beijing, China; ^2^Beijing Institute of Brain Disorders, Capital Medical University, Beijing, China; ^3^Department of Clinical Pharmacy, Dalian Municipal Central Hospital, Dalian, China; ^4^The Russell H. Morgan Department of Radiology and Radiological Sciences, The Johns Hopkins University School of Medicine, Baltimore, MD, USA

## Abstract

**Objective:**

Mesenchymal stem cell (MSC) therapy has been explored in Huntington disease (HD) as a potential therapeutic approach; however, a complete synthesis of these results is lacking. We conducted a meta-analysis to evaluate the effects of MSCs on HD.

**Method:**

Eligible studies published before November 2022 were screened from Embase, PubMed, Web of Science, Medline, and Cochrane in accordance with PRISMA guidelines. ClinicalTrial.gov and the World Health Organization International Clinical Trials Registry Platform were also searched for registered clinical trials. The outcomes in rodent studies evaluated included morphological changes (striatal volume and ventricular volume), motor function (rotarod test, wire hang test, grip strength test, limb-clasping test, apomorphine-induced rotation test, and neuromuscular electromyography activity), cognition (Morris water maze test), and body weight.

**Result:**

The initial search returned 362 records, of which 15 studies incorporating 346 HD rodents were eligible for meta-analysis. Larger striatal and smaller ventricular volumes were observed in MSC-treated animals compared to controls. MSCs transplanted before the occurrence of motor dysfunction rescued the motor incoordination of HD. Among different MSC sources, bone marrow mesenchymal stem cells were the most investigated cells and were effective in improving motor coordination. MSC therapy improved muscle strength, neuromuscular electromyography activity, cortex-related motor function, and striatum-related motor function, while cognition was not changed. The body weight of male HD rodents increased after MSC transplantation, while that of females was not affected.

**Conclusion:**

Meta-analysis showed a positive effect of MSCs on HD rodents overall, as reflected in morphological changes, motor coordination, muscle strength, neuromuscular electromyography activity, cortex-related motor function, and striatum-related motor function, while cognition was not changed by MSC therapy.

## 1. Introduction

Huntington disease (HD) is a neurodegenerative disorder of the central nervous system resulting from a dominantly inherited CAG trinucleotide repeat expansion in exon 1 of the huntingtin (*HTT*) gene that encodes the Huntingtin protein [1]. Pathological changes are characterized by a general shrinkage of the brain and distinct degeneration of the striatum (caudate nucleus and putamen) [2]. Although HD prevalence is only 4-10 individuals per 100,000, it seriously affects the life quality of the patients in many ways, including movement, cognition, and psychological condition, as well as other functional disabilities [3]. Motor defects typically include chorea and loss of coordination. Psychiatric symptoms, such as depression, psychosis, and obsessive-compulsive disorder, are common in HD [4]. Death typically occurs about 20 years after symptom onset [1].

Current therapies for HD are directed at symptom relief, as there are no any disease-modifying therapies. Therapeutic attempts based on pathogenic mechanisms including gene silencing [5], antiapoptosis/caspase inhibition [6], transglutaminase inhibition [7], antioxidative stress [8], upregulating autophagy [9], and physical exercise [10] have been investigated; unfortunately, none of these have met the criteria for clinical translation. For instance, silencing the expression of the mutant *HTT* gene is attractive; however, allele specificity and off-target effects are not fully resolved. Treatment of mouse models with antioxidants was considered to be beneficial [8], whereas trials of creatine for symptomatic patients were disappointing [11].

Stem cell transplantation has gained substantial attention as a potential treatment strategy for neurodegenerative diseases, including HD [12]. Mesenchymal stem cells (MSCs) are superior for their rapid proliferation, lower immunogenicity, and vast sources including bone marrow, adipose tissue, umbilical cord, olfactory mucosa, peripheral blood, placenta, and amniotic fluid [13]. The repair mechanisms of MSCs are mainly attributed to neurotropic, immunoregulatory, antioxidant, and antiapoptotic pathways [14, 15]. Accumulating studies are investigating the effects of MSCs on HD [12, 16–37]; however, a complete synthesis of these results is lacking. We performed a meta-analysis to evaluate the overall effect size to provide objective and comprehensive evidence for the translation of MSC therapy for HD.

## 2. Method

### 2.1. Search Strategy

The literature search was performed according to PRISMA guidelines [38]. Eligible studies published before November 2022 were screened from Embase, PubMed, Web of Science, Medline, and Cochrane. Clinical trials from ClinicalTrials.gov and the World Health Organization International Clinical Trials Registry Platform (WHO ICTRP) were also screened. In Embase, the Emtree terms “mesenchymal stem cell” and “Huntington chorea” and their synonyms were used. The MeSH terms “mesenchymal stem cells” and “Huntington disease” and their synonyms were used in PubMed, Web of Science, Medline, Cochrane, ClinicalTrials.gov, and WHO ICTRP searches. XSL and ZWS (review authors) screened studies for initial inclusion based on titles and abstracts. Full-text screening for eligibility was performed if an initial decision could not be made. In the case XSL and ZWS could not reach a consensus, SL was consulted, followed by discussion and joint consensus in all cases. In addition, other eligible publications selected from the lists of references in the included literature were used to supplement the search results.

### 2.2. Inclusion and Exclusion Criteria

Inclusion criteria were as follows: (1) published as a full-length article in peer-reviewed journals in English; (2) reported quantitative morphological or functional results; (3) used chemical-induced animal models such as the 3-nitropropionic acid (3-NP) induced model, the quinolinic acid (QA) infusion model, and transgenic models including the R6/2, YAC128, and N171-82Q models; and (4) used mesenchymal stem cells, for instance, bone marrow (BM-MSCs)-, amniotic membrane (AMSCs)-, human umbilical cord (UC-MSCs)-, or olfactory ecto (OE-MSCs)-mesenchymal stem cells.

Exclusion criteria were as follows: (1) published as conference abstracts, as reviews, or in retracted papers; (2) reported only *in vitro* results; (3) reported unachievable raw data or did not specify standard deviation (SD) or standard error of mean (SEM); and (4) lacked quantitative data.

### 2.3. Data Extraction

The following information was extracted from the included studies: (1) article information (first author, publication year, journal); (2) animal models (species, sex, type of HD model); (3) MSC treatment modalities (source of MSCs, manipulation of MSCs, MSC passage, age of donors, administration route, doses, number of administrations, follow-up duration); and (4) outcome.

Different outcomes were analyzed including morphological measurements, motor function, cognition, and body weight. For brain morphology, the striatal and ventricular volumes were analyzed, thus reflecting the pathological changes of HD. Functional analyses such as rodent behavior tests were also retrieved. The rotarod test was included to evaluate motor coordination. Muscle strength was analyzed using the grip strength test and the wire hang test. Cortex-related motor function was examined using the limb-clasping test. The apomorphine-induced rotation test was used to evaluate striatum-related motor function. Electrophysiological data was included to reflect neuromuscular electromyography activity. Cognition was analyzed using the Morris water maze (MWM) test. When neurobehavioral tests were performed serially, only the terminal time point data was extracted. If the data was expressed only graphically, raw data was requested from the authors. In the case that the authors did not respond, data was extracted using GetData Graph Digitizer 2.26.

### 2.4. Quality Assessment

The quality of the included studies was assessed independently by XSL and ZWS according to the Collaborative Approach to Meta-Analysis and Review of Animal Data from Experimental Studies (CAMARADES) checklist with minor modifications [39]. One point was given to evidence of each quality criterion: (1) published in a peer-reviewed journal; (2) randomization was used; (3) animals were clearly described ((a) species, (b) background, (c) sex, and (d) age); (4) assessment of behavioral outcomes was blind; (5) transplantation time was clearly stated; (6) the administration route was specified; (7) the doses of MSCs applied were given; (8) pretreatment behavior was assessed; (9) potential conflicts of interest were stated; and (10) suitable animal models were used.

### 2.5. Statistical Analysis

The estimated effect size of MSCs on morphological and functional outcomes of HD rodent models was determined using weighted mean difference (WMD) with a 95% confidence interval (CI) when included studies used the same type of measurements. Otherwise, standardized mean difference (SMD) was analyzed. The statistical significance of the effect size when all studies were pooled was judged by a *Z*-test. A *P* value < 0.05 was considered statistically significant. A leave-one-out sensitivity analysis was performed by iteratively removing one study at a time to confirm whether the findings were driven by any single study.

Potential heterogeneity was initially explored through visual exploration of forest plots. A test for statistical heterogeneity was then performed using Cochrane's *Q*-statistic test (*P* value < 0.1 indicating significance) and *I*^2^ analysis using the following equation:

(1) $$\begin{array}{c} I^{2}=\left(\frac{Q-\text{df}}{Q}\right)\times 100\%, \end{array}$$

where *Q* is the chi^2^ statistic and df is the degree of freedom. Studies with *I*^2^ ≤ 50% were considered to have low heterogeneity; thus, a fixed-effect model was used. Those with *I*^2^ > 50% were considered to have substantial heterogeneity; thus, a random-effect model was adopted. All analyses were done using Review Manager 5.3 software.

## 3. Results

### 3.1. Study Characteristics

**Figure 1:**
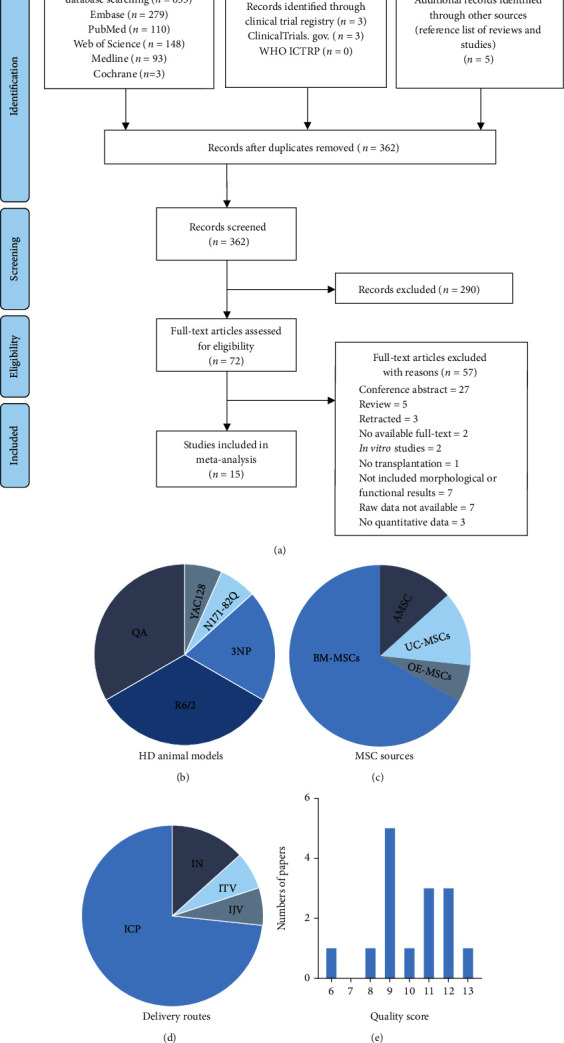
(a) PRISMA flow diagram illustrating the literature search and the studies included. Proportion of (b) HD models, (c) MSC sources, and (d) administration routes used in included studies. (e) Distribution of the methodological quality of these studies.

The initial search returned 362 records, of which 72 were retrieved for full-text review. Fifteen studies including 346 rodent HD animals (145 in the naïve MSC group, 28 in the manipulated MSC group, and 173 in the control group) fulfilled the predefined inclusion criteria and were included in the analysis (Figure 1(a)) [12, 16–29]. The characteristics of included studies are shown in Table 1. All studies used mouse or rat HD models published from 2008 to 2021. Seven studies were performed with transgenic models, six studies used the QA-infusion model, and three used the 3-NP induced model (Figure 1(b)). Transplanted MSCs included BM-MSCs, AMSCs, UC-MSCs, and OE-MSCs (Figure 1(c)). Three studies used manipulated MSCs, including those that induced MSCs into neurotrophic factors secreting cells using special culture medium and those that preconditioned MSCs with lithium and valproic acid. MSCs were infused *via* intranasal, intratail venous, intrajugular venous, and intrastriatal routes (Figure 1(d)). The doses of MSCs ranged from 1 × 10^5^ to 2 × 10^6^. The passage number of MSCs reported ranged from two to eight, whereas two studies did not report the passage number. The age of BM-MSC donors ranged from 6 to 12 weeks old and 2 to 4 months old for mouse and rat donors; however, the age of human donors (adult) was not specified. OE-MSCs were obtained from 20 to 30 years old humans. The extraction of UC-MSCs was performed on P15 mouse pups and newborn human donors. The two AMSC studies did not report the age of donors. The follow-up periods varied from 1 day to 4 months.

### 3.2. Methodological Quality

The methodological quality of included studies is shown in Table 2. The quality scores ranged from 6 to 13 out of a total of 13 points, and the distribution of methodological quality is shown in Figure 1(e). All studies reported the administration routes and MSC doses. Nine studies reported the characteristics of HD models sufficiently, while three studies did not describe sex and four did not report age. Eight studies performed pretreatment behavioral assessment. Six studies reported the randomization of animals into different groups without mentioning a method of randomization. Eleven studies stated potential conflicts of interest.

### 3.3. Effects of MSC Therapy

#### 3.3.1. Brain Morphological Changes

**Figure 2:**
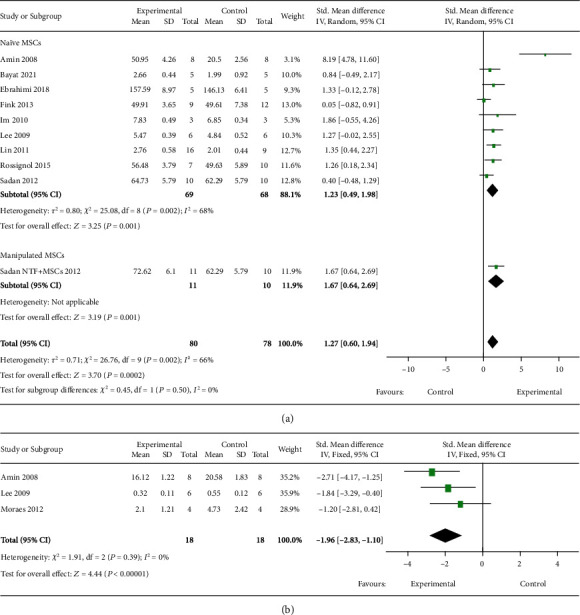
Forest plots of histopathological changes of striatum after MSC therapy for rodent HD models. (a) Striatal volume. (b) Ventricular volume. The sizes of the squares represent the weight that each study contributes to the meta-analysis. The diamond at the bottom represents the overall effect. CI: confidence interval (represented by lines).

The volumes of the striatum and ventricles were measured to evaluate pathological changes, and a beneficial effect was observed with MSC therapy when naïve and manipulated subsets were pooled, although there was substantial heterogeneity (*I*^2^ = 66%, Figure 2(a)). In one study, MSCs were induced to secrete neurotrophic factors using special culture medium and was found to spare the striatum in R6/2 mouse. Nine studies investigating naïve MSCs involving 137 animals reported striatal volume, which included five mouse studies (one QA infusion model and four transgenic models) and four rat studies (two 3-NP induced models and two QA infusion models). Among them, six studies reported striatal volume directly, two reported total area of the brain in pixels, and one study investigated the ratio of the volume of lesioned striatum/contralateral intact striatum. SMD was used. There was high heterogeneity among these studies (*P* = 0.002, *I*^2^ = 68%), which might be explained by one study that did not specify the transplantation time [29]. Removing this study reduced the *I*^2^ value to 7% but did not change the overall result that MSC-treated HD rodents had a larger striatal volume in comparison to the controls (Figure 2(a)). Ventricular volume was measured in three studies. Species difference caused obvious data variation, so SMD was used. The results showed that the ventricular volume of the MSC treatment group was smaller than that in the controls, and no heterogeneity was found between the studies (Figure 2(b)). Altogether, our results show that MSC transplantation improves brain morphological changes in rodent HD.

#### 3.3.2. Motor Coordination

**Figure 3:**
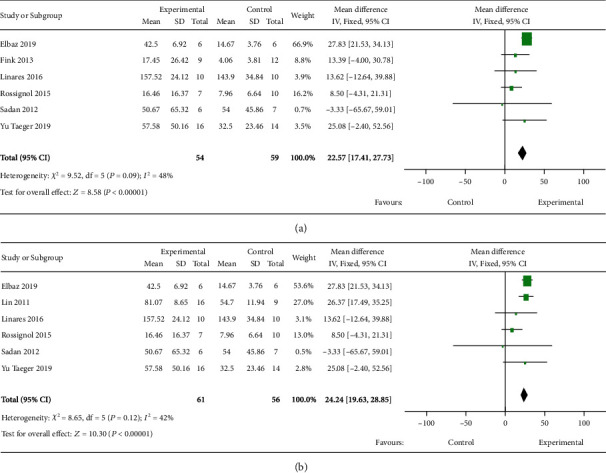
(a) Forest plots of motor coordination tests after early MSC therapy for rodent HD models. (b) Forest plots of motor coordination tests after BM-MSC therapy for rodent HD models. The sizes of the squares represent the weight that each study contributes to the meta-analysis. The diamond at the bottom represents the overall effect. CI: confidence interval (represented by lines).

Motor coordination was analyzed by the latency to fall in the rotarod test in twelve studies. Overall, a beneficial effect was revealed and substantial heterogeneity among studies was found (*I*^2^ = 87%) (Supplementary Figure 1). The two studies that used manipulated MSCs showed high heterogeneity (*I*^2^ = 70%), and motor coordination was not improved by manipulated MSCs (Supplementary Figure 1). Heterogeneity remained high in the ten studies that used naïve MSCs (*I*^2^ = 89%). They revealed significantly improved motor coordination after transplantation (Supplementary Figure 1, detailed study information shown in Table 3). Subgroup analyses of the naïve MSC group were performed according to transplantation time and MSC type to resolve the high heterogeneity. The included studies were separated into early (before motor dysfunction occurrence, six studies) and late (after motor dysfunction occurrence, four studies) transplantation subgroups as the onset of motor dysfunction varies by model. The R6/2 mouse model develops motor dysfunction early at six weeks old [40], and the N171-82Q mouse model occurs after 18 weeks old [41], while the YAC128 mouse model has late onset at 7 months old [30]. A significant improvement in motor coordination was found in the early transplantation group. A relatively low heterogeneity among studies was found (*I*^2^ = 48%) (Figure 3(a)). However, there was a significant heterogeneity (*I*^2^ = 96%) among studies included in the late transplantation group resulting from using different models and cell types, and variations in rotarod speed, posttreatment behavioral test time, and total observation time in the rotarod test. Therefore, these studies were not combined. Detailed study information is described in Table 3.

Studies using the rotarod test were also separated into two subgroups according to the type of naïve MSC transplanted. BM-MSCs showed a significant improvement in coordination with a low heterogeneity (*I*^2^ = 42%, Figure 3(b)). Studies on AMSC transplantation showed high heterogeneity (*I*^2^ = 97%), which was related to the use of different HD models and transplantation time. Details of the AMSC transplantation studies are summarized in Table 3. Studies of other types of MSCs that cannot be combined are also listed in Table 3.

#### 3.3.3. Muscle Strength

**Figure 4:**
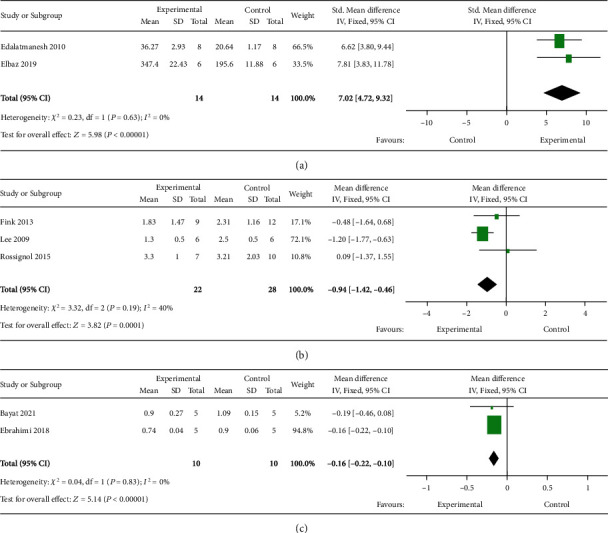
Forest plots of (a) muscle strength, (b) cortex-related motor function, and (c) neuromuscular electromyography activity after MSC therapy for rodent HD models. The sizes of the squares represent the weight that each study contributes to the meta-analysis. The diamond at the bottom represents the overall effect. CI; confidence interval (represented by lines).

Two studies investigated the effects of naïve MSC transplantation on muscle strength in HD models by wire hang and grip strength test. Meta-analysis revealed a positive effect. No heterogeneity between studies was found (Figure 4(a)).

#### 3.3.4. Cortex- and Striatum-Related Motor Function Defects

Three studies performed the limb-clasping test and showed that latency decreased after naïve MSC treatment, indicating an improvement in cortex-related motor function by MSC treatment (Figure 4(b)). There was a low heterogeneity among the studies (*I*^2^ = 40%). Four studies used the apomorphine-induced rotation test to evaluate the striatum-related motor function after stereotactic MSC transplantation into the striatum. An improvement was revealed by the meta-analysis, although the heterogeneity was very high (*I*^2^ = 89%). Substantial heterogeneity was also revealed in studies using naïve MSCs (*I*^2^ = 93%), and no changes were found (Supplementary Figure 2). Detailed study information is described in Table 4.

#### 3.3.5. Neuromuscular Electromyography Activity

Two studies investigated the effect of naïve MSC transplantation on neuromuscular electrophysiological activity. In these experiments, the sciatic nerve was stimulated, and the muscle action potential was recorded in the gastrocnemius muscle. Since no heterogeneity between studies was found, the electromyography latency was analyzed using WMD. The results showed a reduction of latency in the MSC-treated group in comparison with the control group (Figure 4(c)). These results suggest that MSC transplantation improves neuromuscular electromyography performance.

#### 3.3.6. Cognition

MWM tests were performed to assess the cognition of HD rodents after MSC treatment. Three studies analyzed the correction time or the percentage of correction in MWM tests, and a random-effect model was used (*I*^2^ = 85%). The results showed naïve MSC transplantation did not improve the cognition of HD rodents (Supplementary Figure 3). Detailed study information is shown in Table 5.

#### 3.3.7. Body Weight

**Figure 5:**
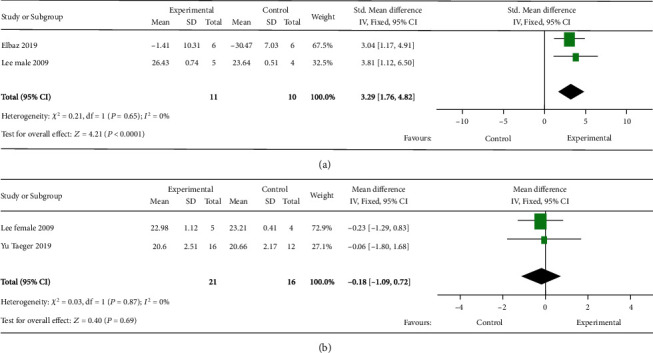
Forest plots of weight in (a) male and (b) female HD mice after MSC therapy. The sizes of the squares represent the weight that each study contributes to the meta-analysis. The diamond at the bottom represents the overall effect. CI: confidence interval (represented by lines).

To investigate the effect of MSC transplantation on body weight, six studies were included. Two studies did not report gender. As significant heterogeneity among studies was found (*I*^2^ = 83%, Supplementary Figure 4), the analysis was then divided into two subgroups according to gender. The studies not reporting gender were excluded. Studies on HD males showed that naïve MSC transplantation increased the body weight (Figure 5(a)). Female studies showed that naïve MSC transplantation did not influence the body weight (Figure 5(b)).

#### 3.3.8. Mechanisms of MSC Therapy for HD Models

Among the included studies, nine investigated potential mechanisms of MSC therapy for HD models, which are summarized in Table 6. Improved neurotrophic function, immune modulation, antiapoptosis, antioxidation, repairment of dopaminergic circuitry, and the promotion of cell proliferation, differentiation, and migration were the proposed mechanisms. Six studies reported factors secreted by the MSCs [20–23, 25, 28], while five studies examined the expression of cytokines in the brains after MSC transplantation [12, 16, 17, 20, 24]. The effect of MSC transplantation on these factors is summarized in Table 7.

### 3.4. Sensitivity Analysis

Sensitivity analysis was performed to evaluate the robustness of the estimated pooled effect sizes for brain morphological changes, motor coordination, and cortex- and striatum-related motor dysfunctions. The pooled effect was stable for brain morphological changes and motor coordination analyses, indicating that these results were not driven by any single study. However, when the study by Lee et al. [28] was removed, statistical significance was lost for the pooled effect size of naïve MSC therapy on cortex-related motor dysfunction, and when removing the study by Sadan et al. [22], naïve MSC treatment showed a beneficial effect on striatum-related motor dysfunction.

## 4. Discussion

**Figure 6:**
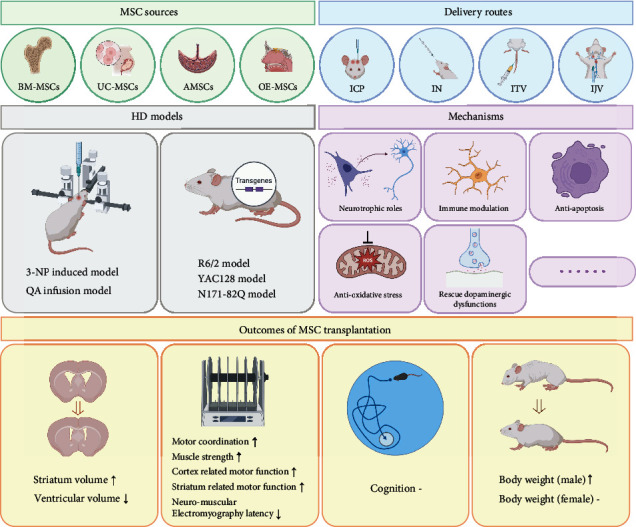
Graphical abstract illustrating the therapeutic outcomes and mechanisms in the meta-analysis of MSC transplantation in HD models.

In recent years, MSC-based therapies for neurodegenerative diseases have gained extensive attention because of their wide spectrum of therapeutic mechanisms involving neurotrophic, immunomodulatory, and regenerative pathways. Diverse MSC types, doses, and administration routes have been investigated in different HD models [12, 16–37] (Figure 6). In this study, we comprehensively collected a wide array of outcome indicators and performed the first meta-analysis on the effects of MSC therapy for HD. Our study reveals that MSC therapy exerts beneficial effects on brain morphology, motor coordination, muscle strength, neuromuscular electromyographical activity, cortex-related motor function, striatum-related motor function, and male-specific body weight gain in HD rodent models. We also showed that cognition was not influenced by MSC therapy. Three clinical trials on MSC therapy for HD (NCT04219241, NCT03252535, NCT02728115) have been registered in ClinicalTrials.gov; however, none have yet reported results; thus, a meta-analysis could not be performed. Detailed information is listed in Supplementary Table 1.

Similar to most cell-based therapies, MSC transplantation may be limited by cell expansion and alteration during long-term culture, the needs of which can vary by route of cell administration and the extent of which can be impacted by the donor and cell source selected. The majority of studies administered MSCs intrastriatally, an efficient method of delivering therapeutic agents to the HD lesion; however, its invasiveness limits its use in clinical settings. As an alternative, intranasal delivery is as a noninvasive method that allows cells to bypass the blood-brain barrier with positive effects in HD [16, 19]. In addition, MSC donor characteristics should also be taken into consideration when designing MSC-based therapies as they can influence MSC isolation, expansion, differentiation, and functional properties *in vitro* [42]. The relationship between donor age and the therapeutic effect is highly complex, as described in detail in the review by Sisakhtnezhad et al. [42].

Data stratification according to the different sources of MSCs revealed that BM-MSCs were the most common MSC source investigated, and it had a positive effect on motor coordination. Regretfully, functional improvement by other sources of MSCs—including UC-MSCs, AMSCs, and OE-MSCs—could not be evaluated by meta-analysis because of the limited number of studies; however, these MSCs have several notable advantages that can compensate for the expansion limitations that currently restrict BM-MSC use: the length of time that they can be cultured and the number of times they can be passaged before senescence. UC-MSCs have a higher harvest rate compared to BM-MSCs [43]. AMSCs are more proliferative than other MSC sources and can be easily isolated from the waste products of liposuction [44]. The benefit of OE-MSCs is that they can be isolated from multiple tissues, such as oral mucosa, tooth tissue, and smell and respiratory mucosa [12]. These advantages have encouraged the use of alternative MSC sources, but more studies are needed to state their benefits more definitively. Two feasible, alternative sources are embryonic stem cells (ESCs) and induced pluripotent stem cells (iPSCs), which can provide an inexhaustible and safe source of MSCs to minimize these issues. Studies have described the application of iPSC- and ESC-derived MSCs in other diseases [45–48], but not yet in HD.

In addition, MSCs have limited capability for self-renewal *in vitro*. MSCs cultured long term and/or with high passage numbers can enter senescence and lose their stem cell characteristics [49]. Serra et al. revealed that passaging human ASCs (up to 12 times) does not significantly influence their secretome, particularly factors that support postnatal neuronal survival, induce neural differentiation, and/or promote axonal growth [50]. However, in HD studies, Rossignol et al. reported R6/2 mice receiving BM-MSCs with high passage numbers (40-50) displayed decreased motor coordination and more morphological deficits than those receiving ones with low passage numbers (3-8), which might be due to less trophic support [20]. Fink et al. reported R6/2 mice receiving UC-MSCs of either high (40-50) or low (3-8) passage number displayed significantly fewer neuropathological deficits and transiently spared spatial memory compared to untreated R6/2 mice [21]. Due to the paucity of studies with high passage numbers, in this meta-analysis, only studies with MSCs of low passage numbers (3-8) were included for comparison.

The studies analyzed in this report consisted of several rodent HD models that confer multiple advantages to the study of HD pathophysiology. Chemically induced HD models—the 3-NP induced model and the QA infusion model—were most often used to evaluate disease progression. The 3-NP induced model leads to metabolic impairment and progressive neurodegeneration of striatal medium spiny neurons, mimicking both the neuropathology and behavioral deficits analogous to those associated with HD [51]. This model was used to analyze both neuromuscular electromyography activity and muscle strength. The QA infusion model, on the other hand, produces behavioral and neuropathological profiles analogous to the early stages of HD [52]. This model was used to evaluate both striatum-related motor function and muscle strength. However, a major limitation of chemical-induced models is the quick development of striatal lesions induced by the chemical compounds only mimicking certain HD symptoms, but not those related to the mutant *HTT* gene; thus, many of the progressive, age-dependent pathogenic events cannot be represented in these acute lesion models. As a result, there is a continuing need for studies to be performed in genetic models including the R6/2, YAC128, and N171-82Q models.

MSCs did not improve the behavior of HD rodents in the MWM test at terminal time points. Whether the progress of recognitive dysfunction could be delayed was unknown because the selection of intermediate points was subjective to the authors and had substantial variation. These results could not be combined and analyzed. In the three studies included, Rossignol et al. demonstrated that BM-MSCs slowed the progressive decline in cognitive performance in the R6/2 mouse model [20]. Edalatmanesh et al. also reported a beneficial effect of BM-MSC transplantation on improving spatial memory deficits in the QA infusion model [27]. Although Fink et al. did not find a significant difference between the MSC-treated R6/2 group and the control R6/2 group, they noticed that cognitive performance in the MSC-treated group did not differ from that in the wild-type group, while R6/2 showed worse behavior than wild-type mice. Thus, the authors suggested an intermediate effect of UC-MSCs on HD-mediated cognitive decline in the R6/2 mouse model in the MWM test at 6 weeks after UC-MSC transplantation [21].

As a familial autosomal dominant disease that can be diagnosed early before symptom onset, therapies for HD can be initiated before major neuronal loss, by which point may be too late for currently available treatments to slow disease progression and to correct neural deficits. In studies that evaluated motor coordination, therapeutic outcome differs by transplantation time. Early transplantation improved the motor coordination of HD rodents, supporting the utility of MSC transplantation as an HD therapeutic. However, whether late MSC transplantation can rescue the motor coordination dysfunction needs more studies to permit a more comprehensive analysis of the role of transplantation time in motor coordination. Replicating these studies, along with studies that elucidate the mechanisms of these cells, will help establish whether there is a critical time window for therapeutic efficacy of MSC transplantation for HD treatment and push it further towards translation.

Although our analysis confirms the utility of MSC transplantation in HD models, the underlying mechanisms remain ambiguous. MSCs can reduce oxidative stress. ASC transplantation activated the CREB signal pathway to upregulate PGC-1*α* and PGC-1*α*-related molecules, including uncoupling protein 2 and 3, superoxide dismutase 1 and 2, and glutathione peroxidase 1, which are all associated with mitochondrial biogenesis and the transcription of molecules that mitigate ROS [28]. In this study, the upregulated expression of mitochondrial and anti-ROS genes potentiated Ca^2+^ homeostasis and reduced the expression levels of both *μ*-calpain and huntingtin fragments [28]. In addition, MSCs can promote cell sparing. BM-MSCs were reported to suppress activation of the Ca^2+^/CaN/NFATc4 pathway to normalize Bax/Bcl2 ratios, regulate Wnt/*β*-catenin signaling, and alleviate aberrant dephosphorylation of *HTT* protein [17, 53, 54]. ASCs can reduce the levels of toxic N-terminal fragments of mutant *HTT* [28]. Yu-Taeger et al. demonstrated increased expression of two markers of dopaminergic signaling in R6/2 mice that received BM-MSC treatment: tyrosine hydroxylase (the rate-limiting enzyme for dopamine biosynthesis) and DARPP-32 (marker of mature medium spiny neurons) [16]. Furthermore, MSCs may also replenish certain cell types. Lin et al. suggested that human BM-MSCs can differentiate into neurons and astrocytes; however, the evidence about differentiation is not strong because the authors only reported the number of cells without proving whether the high numbers were due to differentiation [25]. Lee et al. have also reported initial evidence of AMSC's ability to differentiate into GABAergic neurons *in vivo* in the R6/2 mouse model [28].

Multiple studies have demonstrated cytokine secreted by or induced by MSCs confers some of these therapeutic benefits. With regard to decreased immunity and inflammation, MSC transplantation can reduce the secretion of TNF*α* from the brain of HD mice and upregulate FoxP3 [12, 16, 17]. Furthermore, the reduction of TNF*α* is associated with diminished RIP3, a key inducer of necroptosis [12]. With regard to enhancing neural function, MSCs are also known to secrete regenerative factors, creating a permissive environment for neural progenitor cell migration, as well as axon guidance and elongation [12]. These modulatory actions facilitate axon growth [55] and boost dendrite length [18], which can in turn decrease the inflicted neural area and promote the capacity of neurons to interact with each other [56] to reduce striatal atrophy. The majority of studies we analyzed reported MSCs can secrete and upregulate BDNF in HD [17, 20–23, 26, 28]. The secretion of neurotrophic factors NGF, GDNF, VEGF, HGF, FGF-2, and IGF-1 was also reported [22, 24, 26, 28]. GDNF and VEGF can also help decrease oxidative stress-induced cell death [18]. Increasing evidence shows that MSCs can facilitate extracellular matrix remodeling (e.g., *via* matrix metalloproteinase), which degrades glial scar tissue [12]. Lastly, we showed that MSCs restore changes in brain morphology in HD and that the effects were robust across species, delivery routes, sources of MSCs, and MSC doses, which may suggest a paracrine function of transplanted MSCs as well.

While studies have demonstrated that MSC transplantation could increase the survival rate and prolong the life span of HD rodents [16, 17, 22, 25, 28], adverse events and other safety concerns have yet to be evaluated. The adverse effects of MSCs in clinical trials for other neurological diseases were minor. In trials for stroke, death, stroke recurrence, toxicity related to intravenous infusion, and cell-related serious adverse events were not observed during the 1-year follow-up period [57]. For Alzheimer's disease, commonly occurring events were wound pain from the surgical procedure, fever, dizziness, postoperative delirium, headache, nausea, and vomiting, all of which were alleviated within 36 h or were circumvented with acetaminophen and/or dexamethasone [58, 59]. Major side effects and dose-limiting toxicity did not occur during the 2-year follow-up [58, 59]. For amyotrophic lateral sclerosis, MSC transplantation at times caused modest intercostal pain irradiation and leg sensory dysesthesia, but tumor formation, worsening in psychosocial status, and symptoms of abnormal cell growth were not found in the spinal cord [60, 61]. All the above suggest that this intervention is safe and well tolerated.

Some researchers are now manipulating MSCs pretransplantation to expand their therapeutic benefits. These manipulations could reduce neural damage by releasing factors such as NGF, VEGF, and PIGF-1 *in vitro* [62]. *In vivo* studies have shown that MSCs genetically programmed to overexpress BDNF, induced by special culture medium to secrete neurotrophic factors, and pretreated by lithium and valproic acid improved therapeutic responses [19, 22, 23, 30, 34, 35]. However, due to the limited number of studies and the unavailability of the raw data, we could not conduct a meta-analysis to evaluate their efficacy. Still, we support the development of manipulated MSCs for ultimate use in the clinic.

Others are now using MSCs to augment traditionally acellular therapies. MSCs have been used as carriers to transport drugs and were shown to transport RNAi into HD neurons to reduce *HTT* protein aggregation in cell and organ cultures [63]. Recently, research on MSC-derived exosomes has gained much attention. These exosomes contain a wide range of active molecules [64, 65] and are capable of inducing endogenous neurogenesis and dampen inflammatory responses. Giampà et al. have shown that MSC-conditioned medium can mitigate striatum injury and motor deficits in HD [66]. As MSC-derived exosomes have the advantage of decreased immunogenicity and tumorigenicity compared to MSCs, as well as easy storage, we foresee research efforts shifting to this direction.

We acknowledge there are several limitations to this meta-analysis. Firstly, the sample sizes in some pooled analyses were not large enough—for instance, the analyses on muscle strength, cortex- or striatum-related motor function, neuromuscular electromyography activity, and cognition—having only two to three studies included. More rigorous, larger sample-size preclinical experiments are needed to investigate the therapeutic effects of MSCs. Secondly, several related studies did not state whether their data presentation was in mean ± SD or mean ± SEM and had to be excluded. Their inclusion would have strengthened our meta-analysis. Thirdly, all the studies included were preclinical studies investigating small animal models. Translational and clinical studies were not included because these studies were unreported to date.

## 5. Conclusion

This meta-analysis reveals MSC therapy attenuates morphological changes and improves motor function in HD models, but cognition was not influenced. Furthermore, the weight of male, but not female, HD rodents may be benefited from MSC treatment. These results support MSC-based strategies becoming an alternative treatment for HD; however, before MSC therapies can be translated into clinical practice, their safety, efficacy, and mechanism must be established with more preclinical and clinical studies.

## Figures and Tables

**Table 1:** The characteristics of included studies.

First author and publication year	Journal	Species	Sex	Sample size (MSCs/control)	HD model	Manipulation of MSCs	MSC source	MSC type	Age of donor	Passage of MSCs	MSC dose	Delivery route	Follow-up period	Outcomes
Bayat 2021 [12]	*Journal of Chemical Neuroanatomy*	Wistar rats	M	20 (10/10)	3-NP	None	OE-MSCs-human	Xenogeneic	20- to 30-year-old human	Passage 3	3 × 10^5^	Intra-striatum	30 days	1. Open field 2. Rotarod 3. Muscle activity latency 4. Volume of striatum

Yu-Taeger 2019 [16]	*Cells*	B6CBAF1/J mice	F	32 (16/16)	R6/2	None	BM-MSCs- C57Bl/6	Allogeneic	8- to 12-week-old mice	Passage 3	2 × 10^6^	IN	7.5 weeks	1. Rotarod

Elbaz 2019 [17]	*Neurochemistry International*	Wistar rats	M	24 (12/12)	3-NP	None	BM-MSCs-Wistar	Allogeneic	NR	Passages 2-5	1 × 10^6^	ITV	24 hours	1. Rotarod 2. Grip strength

Ebrahimi 2018 [18]	*Neurotoxicity Research*	SD rats	M	20 (10/10)	3-NP	None	UC-MSCs-human	Xenogeneic	Newborn human	Passage 4	5 × 10^5^	Intra-striatum	30 days	1. Rotarod 2. Muscle activity latency 3. Volume of striatum

Linares 2016 [19]	*Experimental Neurology*	B6C3F1/J mice	M/F	20 (10/10)	N171-82Q	None	BM-MSCs-C57Bl/6	Allogeneic	≤8-week-old mice	Passage 4-7	6 × 10^5^	IN	9 weeks	1. Rotarod
				20 (10/10)		Lithium and valproic acid								1. Rotarod

Rossignol 2015 [20]	*Stem Cell Research & Therapy*	C57BL/6 mice	M/F	17 (7/10)	R6/2	None	BM-MSCs-WT littermates	Syngeneic	6- to 8-week-old mice	Passage 6	4 × 10^5^	Intra-striatum	6 weeks	1. Rotarod 2. Morris water maze 3. Clasping 4. Area of total brain

Fink 2013 [21]	*Stem Cell Research & Therapy*	C57BL/6 mice	M/F	21 (9/12)	R6/2	None	UC-MSCs- C57Bl/6	Allogeneic	15-day-old mice	Passage 3-8	8 × 10^5^	Intra-striatum	6.5 weeks	1. Rotarod 2. Morris water maze 3. Clasping 4. Area of total brain

Sadan 2012 [22]	*Experimental Neurology*	Wistar rats	M	20 (10/10)	QA	None	BM-MSCs-human	Xenogeneic	Adult human	Passage 2-7	1.4 × 10^5^	Intra-striatum	42 days	1. Apomorphine-induced rotation test 2. Volume of striatum
				21 (11/10)		Neurotrophic factors								1. Apomorphine-induced rotation test 2. Volume of striatum

Sadan 2012 [23]	*PLoS Currents Huntington Disease*	C57BL/6 mice	NR	13 (6/7)	R6/2	None	BM-MSCs-human	Xenogeneic	Adult human	Passage 2-6	2 × 10^5^	Intra-striatum	10 weeks	1. Rotarod
				14 (7/7)		Neurotrophic factors								1. Rotarod

Moraes 2012 [24]	*Stem Cell Research*	Wistar rats	M	8 (4/4)	QA	None	BM-MSCs-Wistar	Allogeneic	NR	NR	2 × 10^6^	Intra-striatum	60 days	1. Ventricular area

Lin 2011 [25]	*PLoS One*	C57BL/6 mice	M	25 (16/9)	QA	None	BM-MSCs-human	Xenogeneic	NR	NR	4 × 10^5^	Intra-striatum	13 weeks	1. Volume of striatum 2. Rotarod

Im 2010 [26]	*PLoS Currents Huntington Disease*	C57BL/6 mice	NR	15 (7/8)	YAC128	None	AMSCs-human	Xenogeneic	NR	Passage 3	1 × 10^6^	Intra-striatum	4 months	1. Rotarod 2. Volume of striatum

Edalatmanesh 2010 [27]	*Neurological Research*	Wistar rats	M	16 (8/8)	QA	None	BM-MSCs-Wistar	Allogeneic	3- to 4-month-old rats	Passage 3	5 × 10^5^	IV	9 weeks	1. Apomorphine-induced rotation test 2. Hang wire test 3. Morris water maze

Lee 2009 [28]	*Annals of Neurology*	SD rats	M	12 (6/6)	QA	None	AMSCs-human	Xenogeneic	NR	Passage 5	1 × 10^6^	Intra-striatum	4 weeks	1. Apomorphine-induced rotation test
		C57BL/6 mice	M/F	12 (6/6)	R6/2	None					5 × 10^5^			1. Rotarod 2. Volume of striatum 3. Clasping 4. Ventricular area

Amin 2008 [29]	*Neurological Research*	Wistar rats	NR	16 (8/8)	QA	None	BM-MSCs-Wistar	Allogeneic	2-month-old rats	Passage 3	2 × 10^5^	Intra-striatum	9 weeks	1. Volume of striatum 2. Ventricular area

hUC-MSCs: mesenchymal stem cells from human umbilical cord; BM-MSCs: bone marrow mesenchymal stem cells; OE-MSCs: olfactory ecto-mesenchymal stem cells; AMSCs: mesenchymal stem cells from the amniotic membrane; ITV: intratail venous; IJV: intrajugular venous; IN: intranasal; 3-NP: 3-NP induced model; QA: QA infusion model.

**Table 2:** The methodological quality of enrolled studies.

First author and publication year	1	2	3				4	5	6	7	8	9	10	Quality score
			Species	Background	Sex	Age								
Bayat 2021 [12]	1	1	1	1	1	1	0	1	1	1	1	1	1	12
Yu-Taeger 2019 [16]	1	0	1	1	1	1	0	1	1	1	0	1	1	10
Elbaz 2019 [17]	1	1	1	1	1	0	0	0	1	1	0	1	1	9
Ebrahimi 2018 [18]	1	1	1	1	1	1	0	1	1	1	1	1	1	12
Linares 2016 [19]	1	1	1	1	1	1	1	1	1	1	1	1	1	13
Rossignol 2015 [20]	1	0	1	1	1	1	1	1	1	1	1	1	1	12
Fink 2013 [21]	1	0	1	1	1	1	0	1	1	1	1	1	1	11
Sadan 2012 [22]	1	0	1	1	1	0	0	1	1	1	0	0	1	9
Sadan 2012 [23]	1	1	1	0	0	0	0	0	1	1	0	0	1	6
Moraes 2012 [24]	1	0	1	1	1	1	0	1	1	1	0	0	1	9
Lin 2011 [25]	1	1	1	1	1	1	0	1	1	1	0	1	1	11
Im 2010 [26]	1	0	1	0	0	1	0	1	1	1	1	1	1	9
Edalatmanesh 2010 [27]	1	0	1	1	1	0	0	0	1	1	1	1	1	9
Lee 2009 [28]	1	0	1	1	1	1	0	1	1	1	1	1	1	11
Amin 2008 [29]	1	0	1	1	0	1	0	1	1	1	0	0	1	8

(1) Published in a peer-reviewed journal; (2) randomization was used; (3) detailed animal characteristics were stated ((a) species, (b) background, (c) sex, and (d) age of the animals); (4) blinded assessment of behavioral outcome was used; (5) the specific age at which MSCs were transplanted was stated; (6) the administration route was stated; (7) the number of MSCs was stated; (8) pretreatment behavioral assessment was conducted; (9) potential conflicts of interest were stated; and (10) suitable animal models were used.

**Table 3:** Studies employed rotarod test to evaluate MSC therapy for HD models.

Study	HD model	MSC type	Transplantation time	Post-MSC treatment time (week)	Detection method			Experimental			Control			Statistical significance
					Speed(rpm)	Total observation time (min)	Measurement	Mean	SD	Total	Mean	SD	Total	
Bayat [12]	3-NP induced	OE-MSCs	7 days after the 3-NP injection	4	4-40	5	Latency to fall	227.72	35.85	5	97.19	48.65	5	*P* < 0.05
Elbaz [17]	3-NP induced	BM-MSCs	1 hour before the first 3-NP injection	2	25	Not specified	Latency to fall	42.5	6.92	6	14.67	3.76	6	*P* ≥ 0.05
Fink [21]	R6/2	UC-MSCs	5-week-old	6	10	1	Latency to fall	17.45	26.42	9	4.06	3.81	12	*P* ≥ 0.05
Im [26]	YAC128	AMSCs	12-month-old	4	4-40	3	Latency to fall	67.15	11.68	7	83.21	5.84	8	*P* < 0.05
Lee [28]	R6/2	AMSCs	8.5-week-old	4	4-40	3	Latency to fall	54.19	6.77	6	33.87	4.84	6	*P* < 0.05
Lin [25]	QA infusion	BM-MSCs	7 days after the 3-NP injection	13	30	2	Latency to fall	81.07	8.65	16	54.7	11.94	9	*P* < 0.05
Linares [19]	N171-82Q	BM-MSCs	8-week-old	8	0-30	5	Latency to fall	157.52	24.12	10	143.9	34.84	10	*P* ≥ 0.05
Rossignol [20]	R6/2	BM-MSCs	5-week-old	6	10	1	Latency to fall	16.46	16.37	7	7.96	6.64	10	*P* ≥ 0.05
Sadan [23]	R6/2	BM-MSCs	4-week-old	10	Not specified	4	Latency to fall	50.67	65.32	6	54	45.86	7	*P* ≥ 0.05
Yu-Taeger [16]	R6/2	BM-MSCs	4-week-old	6	4-40	6	Latency to fall	57.58	50.16	16	32.5	23.46	14	*P* = 0.0848

OE-MSCs: olfactory ecto-mesenchymal stem cells; BM-MSCs: bone marrow mesenchymal stem cells; UC-MSCs: mesenchymal stem cells from umbilical cord; AMSCs: mesenchymal stem cells from the amniotic membrane.

**Table 4:** Studies employed apomorphine-induced rotation test to evaluate MSC therapy for HD models.

Study	Posttreatment time (week)	Detection method		Experimental			Control			Statistical significance
		Total observation time (min)	Measurement	Mean	SD	Total	Mean	SD	Total	
Edalatmanesh [27]	5	60	Percentage of net rotations to the ipsilateral hemisphere = (ipsilateral rotations − contralateral rotations)/total rotations × 100	30.72	3.51	8	95.96	8.79	8	*P* < 0.001
Lee [28]	4	60	Net rotations to the ipsilateral hemisphere = ipsilateral rotations − contralateral rotations	14.53	18.9	6	191.86	40.73	6	*P* < 0.01
Sadan [22]	4	45	Net ipsilateral rotations	45.9	44.59	10	69.1	55.02	10	*P* = 0.93

**Table 5:** Studies employed MWM test to evaluate MSC therapy for HD models.

Study	HD model	Posttreatment time (week)	Detection method		Experimental			Control			Statistical significance
			Total observation time (s)	Measurement	Mean	SD	Total	Mean	SD	Total	
Edalatmanesh [27]	QA infusion	9	60	Number of platform crossings	6.52	0.58	8	5.13	0.35	8	*P* < 0.05
Fink [21]	R6/2	6	60	The probability of finding the escape platform = number of correct trials/total trials	0.36	0.35	9	0.13	0.12	12	*P* = 0.13
Rossignol [20]	R6/2	6	60	Correct responses% = (number of correct trials/number of total trials) × 100	16.95	15.3	7	36.36	45.17	10	*P* ≥ 0.05

**Table 6:** Molecular mechanisms of MSC therapy for HD.

Study	Function	Mechanism
Bayat [12]	Antinecroptosis	Downregulating RIP-3, a vitally crucial mediator involved in necroptosis
	Immune regulation	Downregulating the number of activated microglia and the levels of TNF*α*
Yu-Taeger [16]	Immune regulation	Secretion of cytokines and growth factors
		Restoring Iba1 expression and the thickness of the processes of striatum-resident microglia
	Dopaminergic dysfunction repairment	Increasing expression of TH and DARPP-32 protein expressions
Elbaz [17]	Immune regulation	Downregulation of TNF-*α* and FOXP3 levels
		Secretion of VEGF, which can significantly decrease cytosolic Ca^2+^concentration, CaN levels, and NFATc4 expression
	Neurotrophic function	Upregulation of BDNF
	Antiapoptosis	Regulating the Wnt/*β*-catenin signaling pathways
Ebrahimi [18]	Antioxidative stress-induced cell death	Secreting factors such as GDNF and VEGF, decreasing oxidative stress-induced cell death
Rossignol [20]	Neural protection	Secreting BDNF and regulating other NTFs
Sadan [23]	Neural protection	Secreting NTFs
Moraes [24]	Antiapoptosis	Secreting FGF-2 to activate the PI3K/Akt pathway, which is related to cell survival
Lin [25]	Cell proliferation and differentiation	Improving cell proliferation and differentiation, which might be related to chemokine secretion
	Improve cell migration to the injury	Inducing microglia activation and neuroblasts migration into the QA-lesioned region
	Improve angiogenic activity	Integrating with the host cells and increasing the levels of laminin, VWF, SDF-1, and the SDF-1 receptor CXCR4
	Antiapoptosis	Regulating the expression of p-Erk1/2 and Bax
Lee [28]	Antioxidative stress-induced cell death	Increasing the expressions of CREB, PGC-1*α*, and molecules that defend against ROS
	Antiapoptosis	Increasing CREB expression, and secreting soluble factors to reduce the levels of N-terminal fragments of mutant huntingtin

RIP-3: receptor-interacting protein 3; TNF*α*: tumor necrosis factor *α*; VEGF: vascular endothelial growth factor; NFATc4: nuclear factor of activated T cells 4; FOXP3: forkhead box protein P3; GDNF: glial cell derived neurotrophic factor; BDNF: brain-derived neurotrophic factor; NTFs: neurotrophic factors; FGF-2: fibroblast growth factor-2; PI3K: phosphatidylinositol 3′-kinase; VWF: von Willebrand factor; SDF-1: stromal cell-derived factor-1; CXCR4: C-X-C motif chemokine receptor 4; NGF: nerve growth factor; CTNF: ciliary neurotrophic factor; CREB: cAMP response element binding; PGC-1*α*: peroxisome proliferator-activated receptor-gamma coactivator-1*α*; ROS: reactive oxygen species.

**Table 7:** Cytokines secreted and modulated by MSCs in HD.

First author and publication year	MSC source	MSC-secreted factors	Modulated factors in brain (MSCs group vs. control group)
Bayat 2021 [12]	OE-MSCs	/	TNF*α* ↓
Elbaz 2019 [17]	BM-MSCs	/	TNF*α* ↓, FOXP3 ↑, BDNF ↑, NGF -, TrkB -
Yu-Taeger 2019 [16]	BM-MSCs	/	TNF*α* ↓∗, IL -6 ↓∗, CCR5 ↓∗, PTGER2 ↓∗, MCP1 ↓∗, BDNF -, NGF -, VEGF -
Rossignol 2015 [20]	BM-MSCs	BDNF	TNF*α* -
Fink 2013 [21]	UC-MSCs	BDNF	/
Moraes 2012 [24]	BM-MSCs	/	BDNF -, FGF2 ↑
Sadan 2012 [22, 23]	BM/MSCs (neurotrophic factor secreting)	BDNF, GDNF	/
Lee 2011 [28]	ASCs (in the endothelial growth medium/2 MV)	NGF, BDNF, HGF, FGF2, VEGF, IGF1	/
Im 2010 [25]	ASCs (in the endothelial growth medium/2 MV)	NGF, BDNF, HGF, FGF2, VEGF, IGF1	/

BDNF: brain-derived neurotrophic factor, GDNF: glial cell derived neurotrophic factor, NGF: nerve growth factor, HGF: hepatocyte growth factor, FGF2: fibroblast growth factors-2, VEGF: vascular endothelial growth factor, IGF1: insulin-like growth factors-1, TNF*α*: tumor necrosis factor-*α*, FOXP3: forkhead box protein P3, TrkB: tyrosine kinase receptor B, IL-6: interleukin-6, CCR5: C-C chemokine receptor type 5, MCP1: monocyte chemoattractant protein-1, /: not detected, ↓∗ a trend (*P* > 0.05) of decrease reported by the study, -: no significant differences, ↑: significant (*P* < 0.05) increase, ↓: significant (*P* < 0.05) decrease.

## Data Availability

Data will be provided to other investigators upon request made to the corresponding author (SL) in accordance with the International Committee of Medical Journal Editors requirements.

## Supplementary Materials

Supplementary Materials Supplementary Figures 1–4 and Supplementary Table 1 were provided as supplementary materials.
