# Supplementary material for: Mesenchymal Stem Cell Therapy for Huntington Disease: A Meta-Analysis
Source: Stem Cells Int. 2023 Apr 30;2023:1109967. doi: 10.1155/2023/1109967 (PMC10164866; doi:10.1155/2023/1109967)
Supplement: Supplementary Materials — Supplementary Figures 1–4 and Supplementary Table 1 were provided as supplementary materials. [file 1109967.f1.docx]

**
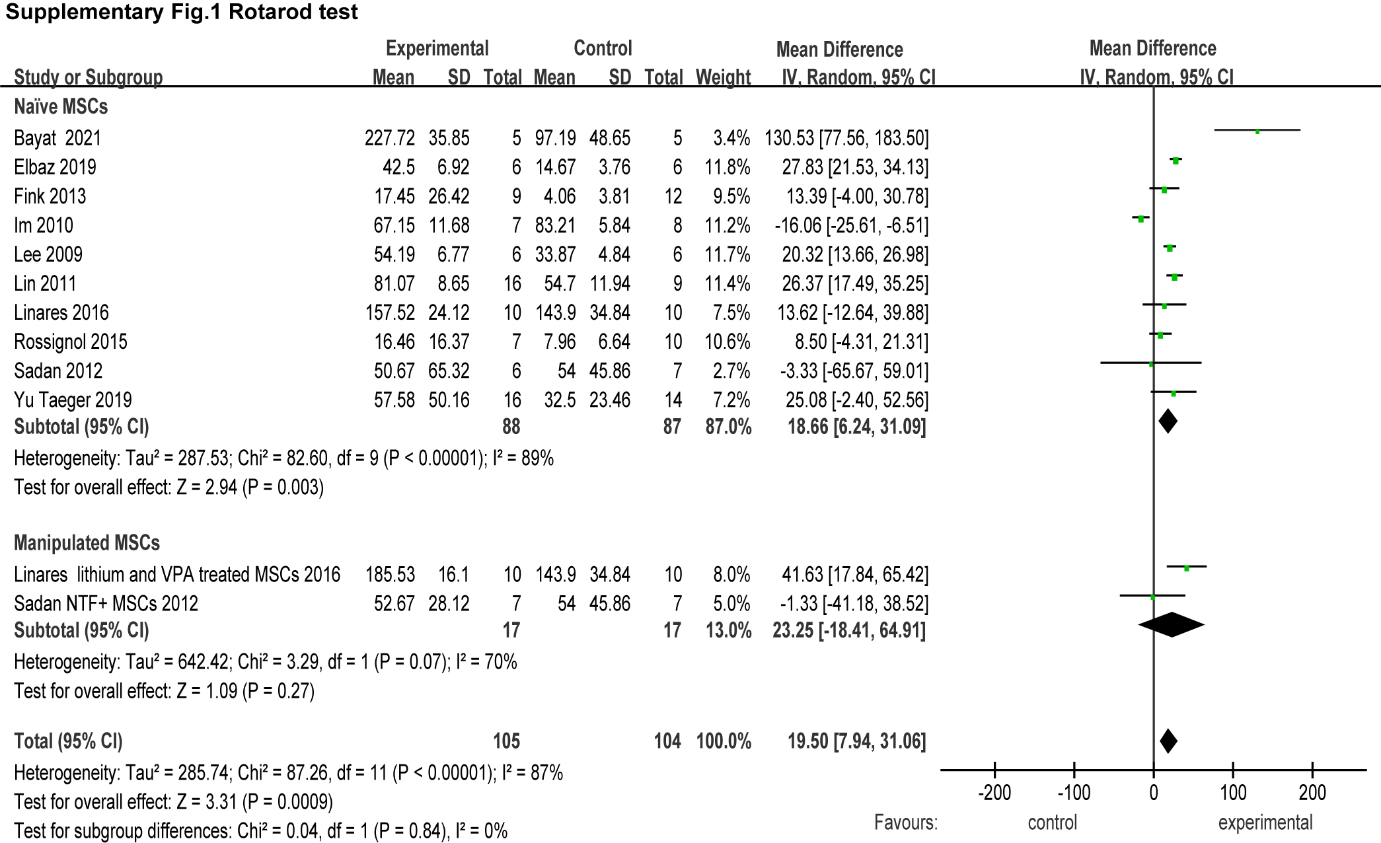
**

**Supplementary Fig.1.** Forest plots of motor coordination after MSC-therapy for rodent HD models. The sizes of the squares represent the weight that each study contributes to the meta-analysis. The diamond at the bottom represents the overall effect. CI = confidence interval (represented by lines).

**
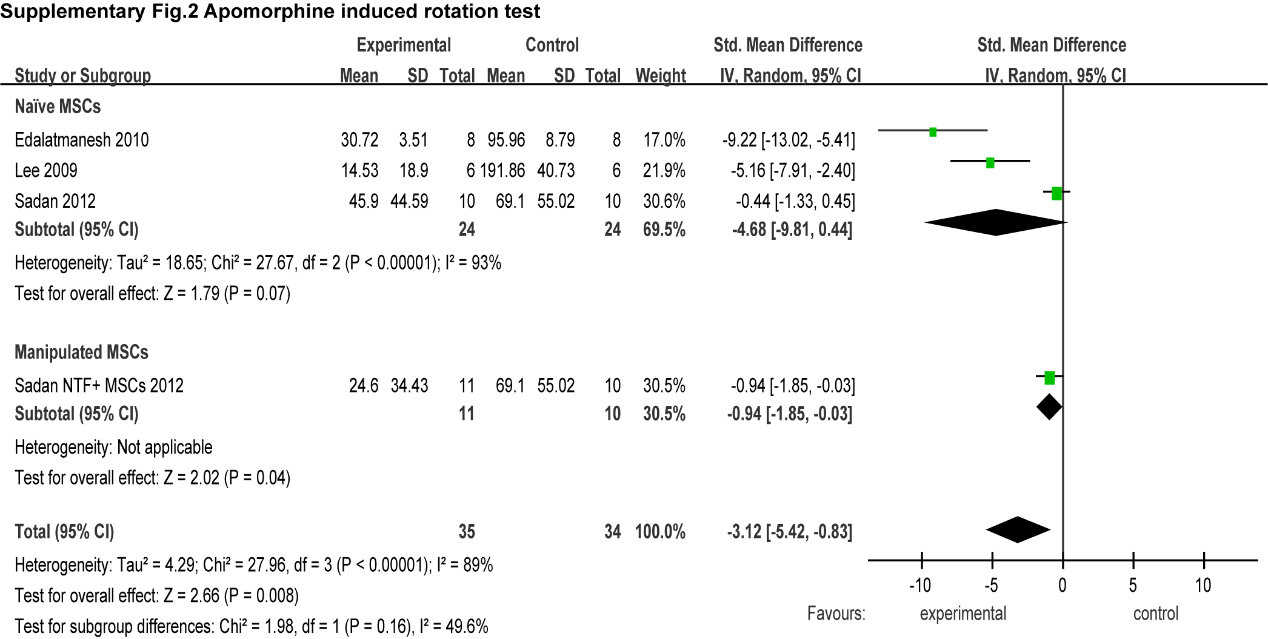
**

**Supplementary Fig.2.** Forest plots of the apomorphine induced rotation test after MSC-therapy for rodent HD models. The sizes of the squares represent the weight that each study contributes to the meta-analysis. The diamond at the bottom represents the overall effect. CI = confidence interval (represented by lines).

**
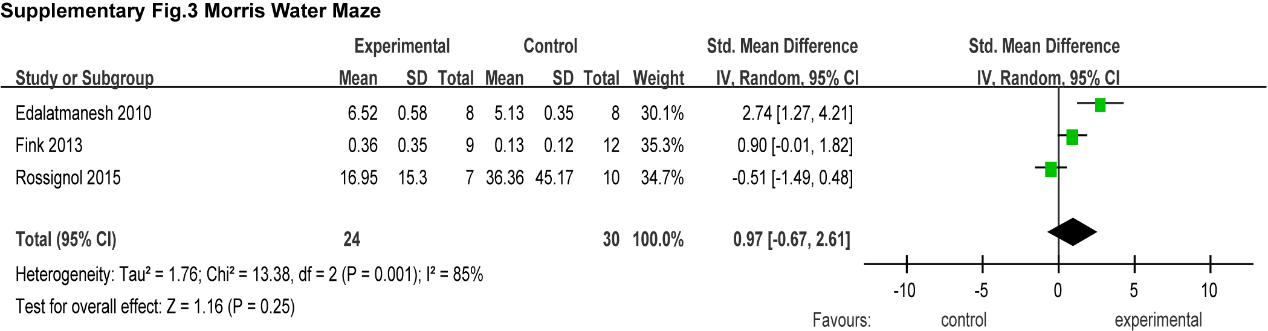
**

**Supplementary Fig.3.** Forest plots of cognitive function after MSC-therapy for rodent HD models. The sizes of the squares represent the weight that each study contributes to the meta-analysis. The diamond at the bottom represents the overall effect. CI = confidence interval (represented by lines).

**
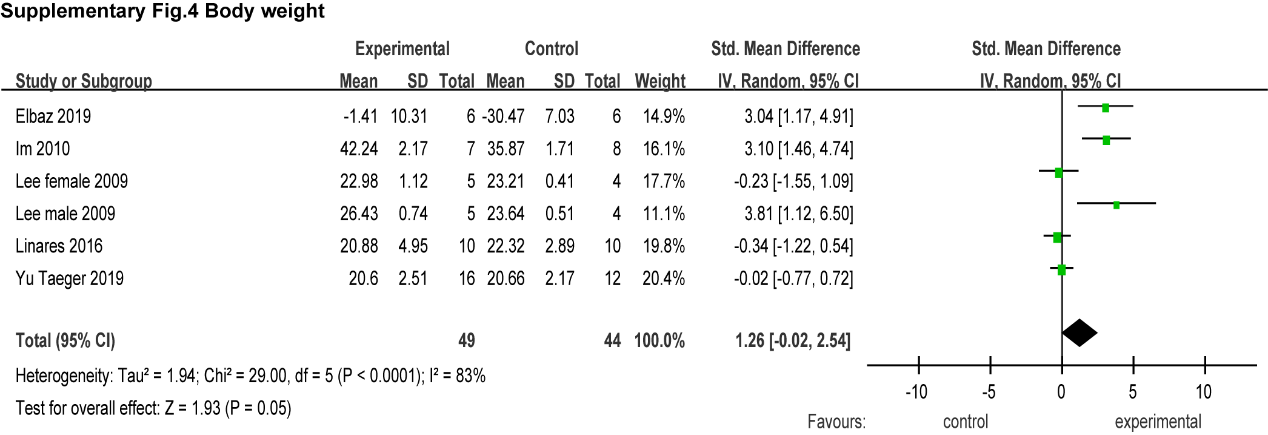
**

**Supplementary Fig.4.** Forest plots of body weight after MSC-therapy for rodent HD models. The sizes of the squares represent the weight that each study contributes to the meta-analysis. The diamond at the bottom represents the overall effect. CI = confidence interval (represented by lines).

**Supplementary Table 1** Clinical trials for MSC transplantation in HD registered at Clinicaltrials.gov (accessed November 2022).

| **NCT Number** | **Title** | **Status** | **Interventions** | **Phase** | **Allocation** | **Masking** | **Outcome Measures** | **Enrollment** | **Age** | **Sex** | **Dates** | **Locations** |
| --- | --- | --- | --- | --- | --- | --- | --- | --- | --- | --- | --- | --- |
| NCT04219241 | Clinical Extension Study for Safety and Efficacy Evaluation of Cellavita-HD Administration in Huntington's Patients. | Active, not recruiting | •Biological: Cellavita-HD | II/III | N/A | Open Label | Efficacy  •Clinical progression of the disease  •Clinical neurological worsening over the treatment by UHDRS  •BMI assessment  •CNS assessment by MRI  Safety  •Risk of suicidal ideation and/or behavior by C-SSRS  •Risk of suicidal ideation and/or behavior by HDS | 35 | 21 Years - 65 Years | All | February 10, 2020-October 28, 2022 | Azidus Brasil Pesquisa Científica e Desenvolvimento Ltda., Valinhos, São Paulo, Brazi |
| NCT03252535 | Dose-response Evaluation of the Cellavita HD Product in Patients with Huntington's Disease | Completed | •Biological: Cellavita HD lower dose  •Biological: Cellavita HD higher dose  •Other: Placebo | II | Randomized | Triple (Participant, Investigator, Outcomes Assessor) | Efficacy  •Effective dose  •Clinical neurological worsening over the treatment  •BMI assessment  •Risk of suicidal ideation  •CNS assessment  •CIBIS  Safety  •Effective dose | 35 | 21 Years - 65 Years | All | January 15, 2018- October 28, 2022 | Azidus Brasil Pesquisa Científica e Desenvolvimento Ltda., Valinhos, São Paulo, Brazil |
| NCT02728115 | Safety Evaluation of Cellavita HD Administered Intravenously in Participants with Huntington's Disease | Active, not recruiting | •Biological: Cellavita HD lower dose  •Biological: Cellavita HD higher dose | I | Non-Randomized | Open Label | Efficacy (Preliminary)  •UHDRS  •Global clinical response by CIBIS  •Comparison of the inflammatory markers  •Preliminary efficacy of Cellavita HD by comparison of the CNS assessment  Safety  •Periodic monitoring of adverse events, vital signs, laboratory tests, ECG and incidence of benign and malignant neoplasms  •Risk of suicidal ideation by HDRS  Mechanism  •Immunological response of Cellavita HD | 6 | 21 Years - 65 Years | Male | October 16, 2017-November 2, 2022 | Azidus Brasil Pesquisa Científica e Desenvolvimento Ltda., Valinhos, São Paulo, Brazil |

Abbreviations: UHDRS: unified Huntington disease rating scale; BMI: body mass index; CNS: central nervous system; MRI: magnetic resonance imaging; C-SSRS: Columbia-suicide severity rating scale; HDS: Hasegawa’s dementia scale; CIBIS: clinical interview-based impression of severity; ECG: electrocardiogram; HDRS: Hamilton depression rating scale.
